# Roscovitine Attenuates Microglia Activation and Monocyte Infiltration via p38 MAPK Inhibition in the Rat Frontoparietal Cortex Following Status Epilepticus

**DOI:** 10.3390/cells8070746

**Published:** 2019-07-19

**Authors:** Ji-Eun Kim, Hana Park, Seo-Hyeon Choi, Min-Jeong Kong, Tae-Cheon Kang

**Affiliations:** 1 Department of Anatomy and Neurobiology, College of Medicine, Hallym University, Chuncheon 24252, Korea; 2 Institute of Epilepsy Research, College of Medicine, Hallym University, Chuncheon 24252, Korea

**Keywords:** CCR2, CD68, epilepsy, IB4, Iba-1, LAMP1, NFκB, SB202190, seizure

## Abstract

Under physiological conditions, microglia are unique immune cells resident in the brain that is isolated from the systemic immune system by brain-blood barrier. Following status epilepticus (SE, a prolonged seizure activity), microglia are rapidly activated and blood-derived monocytes that infiltrate the brain; therefore, the regulations of microglia activation and monocyte infiltration are one of the primary therapeutic strategies for inhibition of undesirable consequences from SE. Roscovitine, a potent (but not selective) cyclin-dependent kinase 5 (CDK5) inhibitor, has been found to exert anti-inflammatory and microglia-inhibiting actions in several in vivo models, although the underlying mechanisms have not been clarified. In the present study, roscovitine attenuated SE-induces monocyte infiltration without vasogenic edema formation in the frontoparietal cortex (FPC), accompanied by reducing expressions of monocyte chemotactic protein-1 (MCP-1) and lysosome-associated membrane protein 1 (LAMP1) in resident microglia, while it did not affect microglia transformation to amoeboid form. Furthermore, roscovitine ameliorated the up-regulation of p38 mitogen-activated protein kinase (p38 MAPK) phosphorylation, but not nuclear factor-κB-S276 phosphorylation. Similar to roscovitine, SB202190, a p38 MAPK inhibitor, mitigated monocyte infiltration and microglial expressions of MCP-1 and LAMP1 in the FPC following SE. Therefore, these findings suggest for the first time that roscovitine may inhibit SE-induced neuroinflammation via regulating p38 MAPK-mediated microglial responses.

## 1. Introduction

Under physiological conditions, microglia are unique immune cells resident in the brain that is isolated from the systemic immune system by a brain-blood barrier. Under pathological conditions, microglia are rapidly activated, and increase synthesis and release of various cytokines and chemokines in the brain [1,2,3,4,5]. Therefore, microglia activation is one of the common and early hallmarks of neurological diseases. Status epilepticus (SE) is defined as continuous unremitting seizure activity longer than 5 min, which is a medical emergency with significant mortality and one of the epileptogenic insults [6]. SE results in specific patterns of neuron loss and astroglial/microglial responses in various brain regions. In particular, SE-induced microglia activation accelerates vasogenic edema and neuronal death [7,8]. Furthermore, SE leads to blood-derived leukocyte infiltration in brain parenchyma. Briefly, neutrophils transiently invade brain parenchyma during the acute phase of SE, disappearing thereafter. Later, monocytes infiltrate and persist during the epileptogenic period [7,9]. Therefore, the regulations of microglia activation and monocyte infiltration are one of the primary therapeutic strategies for inhibition of undesirable consequences from SE.

Although the underlying mechanisms have not been clarified, SE induces monocyte infiltration without vasogenic edema formation in the frontoparietal cortex (FPC) by up-regulation of monocyte chemotactic protein-1 (MCP-1) in activated microglia. MCP-1 is primarily credited with recruitment of macrophage populations to sites of expression mediated by its receptor, C-C motif chemokine receptor 2 (CCR2) [7,10,11]. Interestingly, roscovitine, a non-selective cyclin-dependent kinase 5 (CDK5) inhibitor, has been found to exert anti-inflammatory and microglia-inhibiting actions in several in vivo models [12,13,14]. Thus, it is likely that roscovitine may attenuate SE-induced monocyte infiltration by inhibiting microglia activation, which has not been examined, and this issue is the focus of the present study.

Here, we demonstrate for the first time that roscovitine effectively ameliorated SE-induced microglia activation and monocyte infiltration by inhibiting MCP-1 induction in activated microglia, which was mediated by p38 mitogen-activated protein kinase (p38 MAPK) activation independent of nuclear factor-κB (NFκB)-S276 phosphorylation. Therefore, our findings suggest that roscovitine may attenuate SE-induced neuroinflammation via regulating microglia-mediated responses.

## 2. Materials and Methods

### 2.1. Experimental Animals and Chemicals

The present study was carried out on adult male Sprague-Dawley (SD) rats (7 weeks old). Animals were housed in a controlled room temperature (22 ± 2 °C), humidity (55 ± 5%) and a light-dark cycle on a 12-h on-off cycle. Food and water were available ad libitum throughout the experiments. All experimental protocols described below were approved by the Institutional Animal Care and Use Committee (Hallym 2018-2, April, 2018) of Hallym University (Chuncheon, Korea). Every effort was made to reduce the number of animals employed and to minimize animal discomfort. All reagents were obtained from Sigma-Aldrich (St. Louis, MO, USA), except as noted.

### 2.2. Surgery and Drug Infusion

Roscovitine penetrates the brain-blood barrier (BBB), but is rapidly eliminated from brains in adult rats (<30 min) [15,16]. Thus, it is likely that the half-life of roscovitine concentration in the brain would be too short to maintain its pharmacological actions. In addition, it could not be excluded that systemic roscovitine treatment would affect peripheral (blood) monocytes, which would influence monocyte infiltration in the brain following SE. Therefore, we have chosen brain infusion of roscovitine rather than its systemic administration in order to maintain roscovitine concentration in the brain and to avoid its peripheral effects. Surgery for drug infusion was performed as previously described [17,18,19]. Briefly, rats were anesthetized with Isoflurane anesthesia (3% induction, 1.5–2% for surgery and 1.5% maintenance in a 65:35 mixture of N_2_O:O_2_) and placed in a stereotaxic frame. A brain infusion kit 1 (Alzet, Cupertino, CA, USA) was implanted into the right lateral ventricle (1 mm posterior; 1.5 mm lateral; 3.5 mm depth) and connected to an osmotic pump (1007D, Alzet, Cupertino, CA, USA) containing (1) vehicle; (2) roscovitine (100 μM); or (3) SB202190 (a p38 MAPK inhibitor; 0.3 mg/mL). Each compound was infused over 7 days, since at least a 3-day infusion before SE induction shows the pharmacological effects of roscovitine on events induced by SE [17,18]. In addition, 3-days after SE is the best time point to validate the effect of roscovitine on both microglia activation and monocyte infiltration [7]. In a pilot study and our previous studies [17,18,19], each treatment did not show behavioral and neurological defects and could not change the seizure susceptibility and seizure severity in response to pilocarpine in normal animals.

### 2.3. SE Induction

Three days after surgery, rats were treated with pilocarpine (380 mg/kg, i.p.) as previously described [17,18,19]. Atropine methylbromide (5 mg/kg, i.p.) was injected 20 min before a single dose of pilocarpine in order to avoid the peripheral muscarinic effect. To terminate SE, diazepam (Valium; Hoffman la Roche, Neuilly sur-Seine, France; 10 mg/kg, i.p.) was administered 2 h after onset of SE and repeated as needed. As controls, age-matched normal rats were treated with saline instead of pilocarpine. 

### 2.4. Tissue Processing and Immunohistochemistry

Under urethane anesthesia (1.5 g/kg, i.p.), animals were perfused via a cannula into the left ventricle of the heart with 0.9% saline, followed by 4% paraformaldehyde in a 0.1 M phosphate buffer (PB, pH 7.4). After perfusion, the brains were removed and post-fixed in the same fixative overnight, subsequently cryoprotection with 30% sucrose/0.1M PBS. Brain coronal sections of 30 μm were obtained with a cryo-microtome. Free-floating sections were washed 3 times in PBS (0.1M, pH 7.3). Next, to deactivate the endogenous peroxidase, sections were incubated in 3% H_2_O_2_ and 10% methanol in PBS (0.1M) for 20min at room temperature. Later, sections were incubated in primary antibody (Table 1). Tissue sections were developed in 3,3′-diaminobenzidine in 0.1 M Tris buffer and mounted on gelatin-coated slides. Some sections were incubated with a cocktail solution containing the primary antibodies or isolectin B4 (IB4, Table 1) in PBS containing 0.3% Triton X-100 overnight at room temperature. Thereafter, sections were visualized with appropriate Cy2- and Cy3-conjugated secondary antibodies. Immunoreaction was observed using an Axio Scope microscope (Carl Zeiss Korea, Seoul, Korea). To establish the specificity of the immunostaining, a negative control test was carried out with preimmune serum instead of the primary antibody. No immunoreactivity was observed for the negative control in any structures (data not shown). All experimental procedures in this study were performed under the same conditions and in parallel.

### 2.5. Cell Count and Measurement of Iba-1 Positive Area

The number of cells was measured, as previously described [7]. Briefly, sections (10 sections per each animal) were captured using an AxioImage M2 microscope, and areas of interest (1 × 10^4^ μm^2^) were selected from the FPC. Thereafter, ionizing calcium-binding adaptor molecule 1 (Iba-1) positive area was measured by using AxioVision Rel. 4.8 software. Cells were also counted on 20× images using AxioVision Rel. 4.8 Software. Cell counts were performed by two different investigators who were blind to the classification of tissues.

### 2.6. Data Analysis

All data obtained from the quantitative measurements were analyzed using Student *t*-test and one-way ANOVA to determine statistical significance. Bonferroni’s test was used for post hoc comparisons. A *p*-value below 0.05 was considered statistically significant.

## 3. Results

### 3.1. The Effects of Roscovitine on Microglia Activation and Monocyte Infiltration Following SE

**Figure 1:**
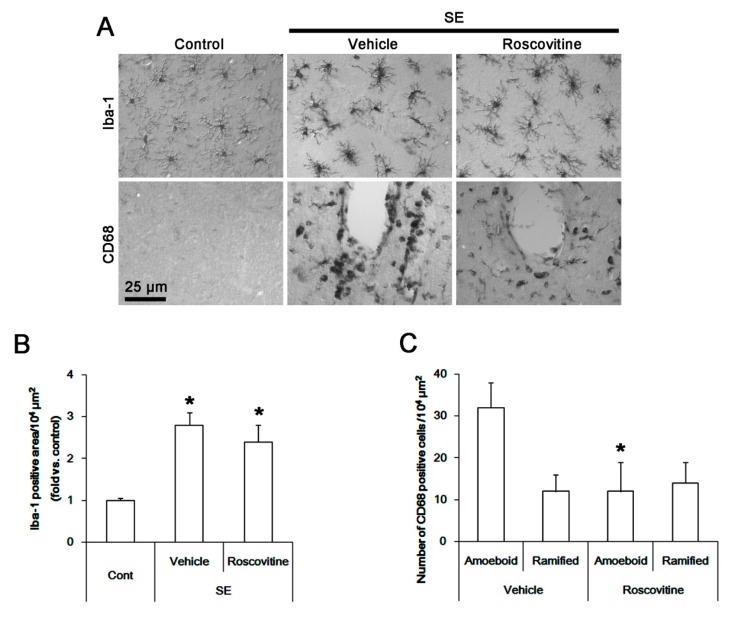
**The effect of roscovitine on microglia activation and monocyte infiltration in FPC following SE**. Iba-1 microglia show hypertrophic morphologies with hyper-ramified processes that are covered by a lot of thorny spine following SE. In addition, CD68 cells showing amoeboid or round shapes are detected in the FPC following SE. These CD68 cells are localized in perivascular brain parenchyma. A few CD68 cells exhibited hyper-ramified shapes. Although roscovitine does not influence Iba-1 microglia transformation, it reduces the number of CD68 amoeboid cells without altering that of CD68 hyper-ramified cells. (A) Representative images for Iba-1 and CD68 positive cells. (B,C) Quantification of the effect of roscovitine on Iab-1 positive area (**B**) and the number of CD68 amoeboid and ramified cells (**C**) following SE. Error bars indicate SEM (* p < 0.05 vs. control- or vehicle-treated animals; *n* = 7, respectively).

In control animals, Iba-1 microglia showed a slender ramified and stellate appearance indicative of resting cells in the FPC (Figure 1A). However, CD68 cells were rarely observed in this region (Figure 1A). Following SE, Iba-1 microglia showed hypertrophic morphologies with irregularly shaped soma and blunted processes that were covered by many thorny spines (Figure 1A). These indicate the activated states of microglia [20]. Thus, the Iba-1 positive area was increased, as compared to control animals (*p* < 0.05, one-way ANOVA, *n* = 7, respectively; Figure 1B). In addition, CD68 cells showing amoeboid or round shapes with smooth cell surface (Figure 1A) which indicate phagocytic and cytotoxic activity [20] were detected in the FPC following SE. These CD68 cells were localized in perivascular brain parenchyma. A few CD68 cells exhibited hyper-ramified shapes (Figure 1A). Although roscovitine did not influence the Iba-1 microglia transformation (Figure 1B), it resulted in ~ 38% reductions in the number of CD68 amoeboid/round cells without altering that of CD68 hyper-ramified cells in the FPC (*p* < 0.05 vs. vehicle, Student *t*-test, *n* = 7, respectively; Figure 1C). Thus, the fraction of hyper-ramified cells in CD68 cells was higher than those in amoeboid or round cells in roscovitine-treated animals, as compared to vehicle-treated animals. Since CD68 and Iba-1 are commonly used markers for peripheral monocytes/activated microglia and resident microglia/activated microglia, respectively [7,21], these findings suggest that roscovitine may mitigate monocyte infiltration into the brain parenchyma, but not morphogenesis of activated microglia, independent of vasogenic edema formation following SE.

### 3.2. The Effect of Roscovitine on Microglial MCP-1 Induction After SE

**Figure 2:**
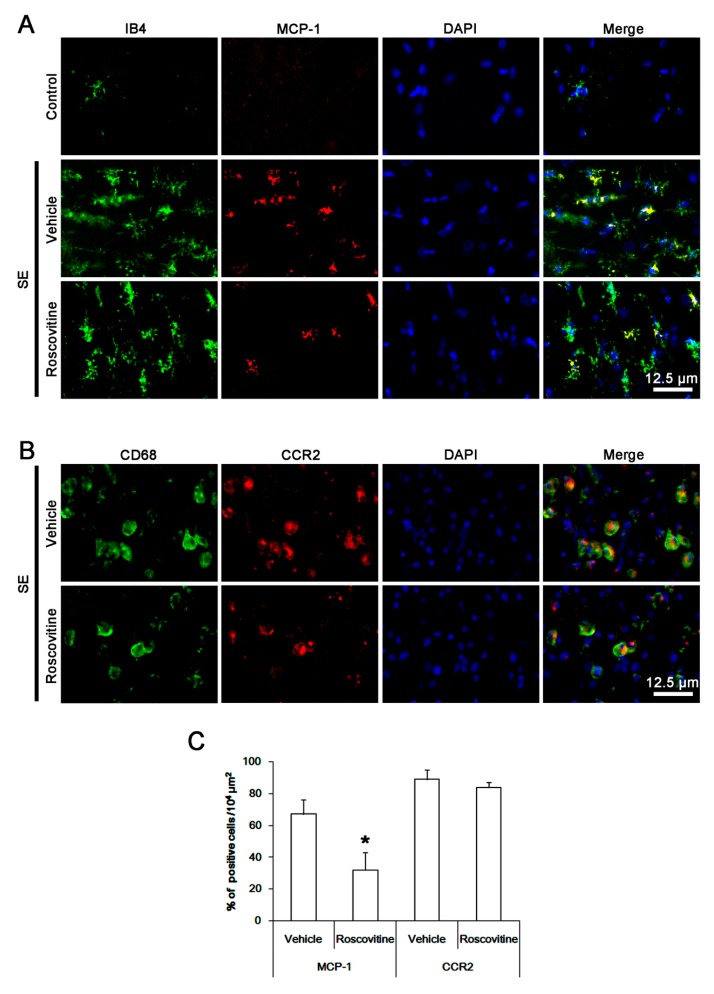
**The effect of roscovitine on MCP-1 induction after SE**. Three days after SE, MCP-1 expression is increased in resident IB4 microglia in vehicle-treated animals, while it is rarely detected under physiological condition. CD68 cells show CCR2 (receptor for MCP-1) expression. Following SE, roscovitine diminishes MCP-1 expression in IB4 microglia. However, CCR2 expression in CD68 cells is unaffected by roscovitine. (A,B) Representative images for MCP-1 and CCR2 expression in IB4 microglia and CD68 cells, respectively. (C) Quantification of the effect of roscovitine on MCP-1 and CCR2 expression in IB4 microglia and CD68 cells following SE. Error bars indicate SEM (* p < 0.05 vs. vehicle-treated animals; *n* = 7, respectively).

MCP-1 is required for efficient monocytes recruitment into brain parenchyma [11]. Indeed, the up-regulation of MCP-1 in activated microglia induces monocyte infiltration following SE [7]. Therefore, we performed the double immunofluorescent study for MCP-1 and IB4 (a lectin marker for microglia) instead of Iba-1 to evaluate the effect of roscovitine on this chemokine following SE. MCP-1 expression was increased in activated IB4 microglia following SE, while it was rarely detected under physiological condition (Figure 2A). CD68 cells showed CCR2 (receptor for MCP-1) expression (Figure 2B). Roscovitine effectively diminished MCP-1 expression in IB4 microglia, thus the fraction of MCP-1 positive cells in IB4 microglia was reduced to 48%, as compared to vehicle (*p* < 0.05 vs. vehicle, Student *t*-test, *n* = 7, respectively; Figure 2A,C). However, CCR2 expression in CD68 cells was unaffected by roscovitine (Figure 2B,C). These findings indicate that roscovitine may ameliorate blood-derived monocyte infiltration via inhibiting MCP-1 induction in activated microglia following SE. 

### 3.3. The Effects of Roscovitine on Phagocytosis of Activated Microglia and Monocytes Following SE

**Figure 3:**
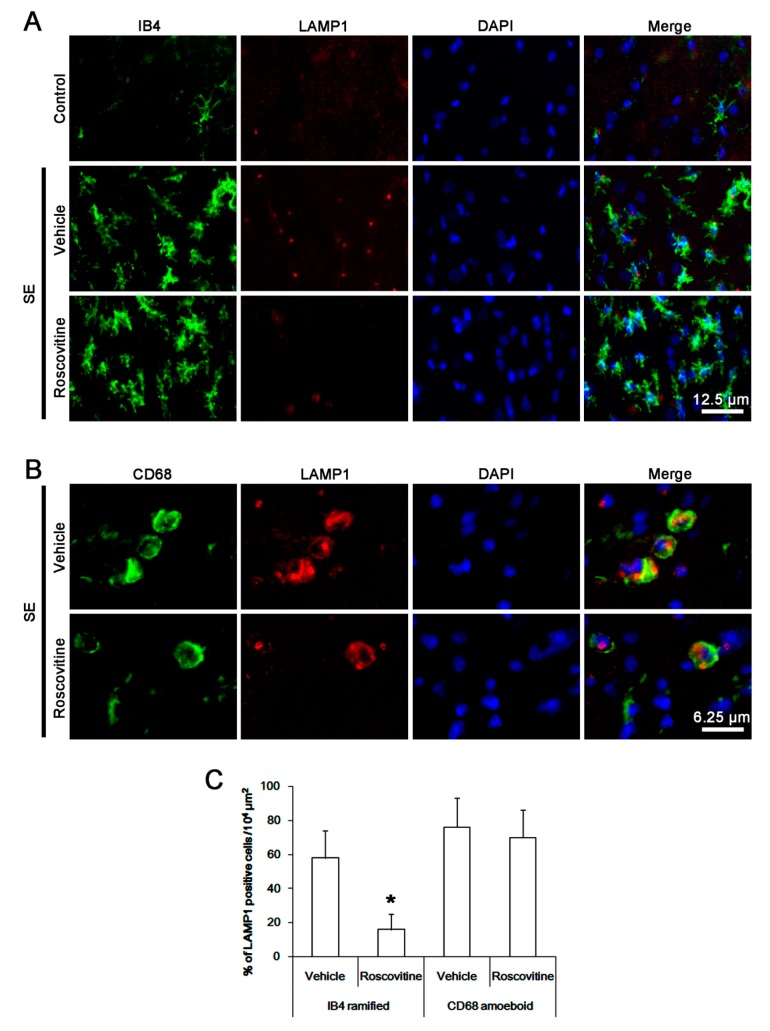
**The effect of roscovitine on phagocytosis of resident microglia and monocyte following SE**. Three days after SE, LAMP1 expression is significantly increased in resident IB4 ramified microglia and CD68 amoeboid cells in vehicle-treated animals, while it is rarely detected under physiological condition. Following SE, roscovitine alleviates the up-regulation of LAMP1 expression in IB4 ramified microglia, but not in CD68 amoeboid cells. (A,B) Representative images for LAMP1 expression in IB4 microglia and CD68 cells, respectively. (C) Quantification of the effect of roscovitine on LAMP1 expression in IB4 microglia and CD68 cells following SE. Error bars indicate SEM (* p < 0.05 vs. vehicle-treated animals; *n* = 7, respectively).

Next, we investigated whether roscovitine influences the phagocytic abilities of activated microglia and blood-derived monocytes following SE. Compared to control animals, lysosome-associated membrane protein 1 (LAMP1, a marker for activation of phagocytosis) [22] expression was significantly increased in activated IB4 ramified microglia and CD68 amoeboid cells (Figure 3A,B). Thus, 58 and 76% of IB4- and CD68 cells showed LAMP1 expressions (Figure 3C). In particular, the up-regulation of LAMP1 expression was more predominant in CD68 cells than that of IB4 microglia (Figure 3A,B). Roscovitine alleviated the up-regulation of LAMP1 expression in IB4 ramified microglia following SE. Thus, the fraction of LAMP1 cells in IB4 cells was reduced to 16% (*p* < 0.05 vs. vehicle, Student *t*-test, *n* = 7, respectively; Figure 3A–C). However, roscovitine did not affect LAMP1 expression in CD68 amoeboid cells following SE. Therefore, our findings indicate that roscovitine may also inhibit activation and phagocytosis of activated resident microglia, but not blood-derived monocyte following SE.

### 3.4. The Effects of Roscovitine on NFκB-S276 Phosphorylation in Microglia and Monocytes

**Figure 4:**
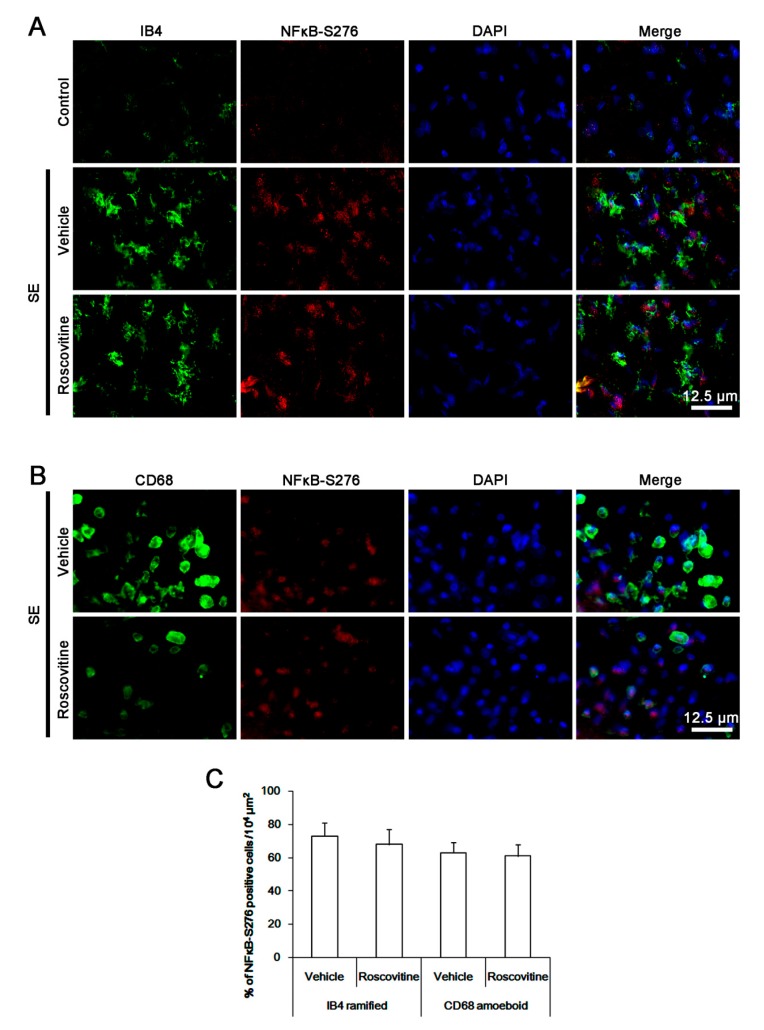
**The effect of roscovitine on NFκB-S276 phosphorylation in microglia and monocyte following SE**. NFκB-S276 phosphorylation is apparently increased in most of resident IB4 microglia and CD68 cells following SE, while it is rarely observed in control animals. Roscovitine does not affect p65 NFκB-S276 phosphorylation in resident IB4 microglia and CD68 cells (A,B) Representative images for NFκB-S276 phosphorylation in IB4 microglia and CD68 cells, respectively. (C) Quantification of the effect of roscovitine on NFκB-S276 phosphorylation in IB4 microglia and CD68 cells following SE. Error bars indicate SEM (* p < 0.05 vs. vehicle-treated animals; *n* = 7, respectively).

p65 NFκB-S276 phosphorylation is important for MCP-1 production in macrophages, microglia activation and monocyte infiltration [23,24]. To elucidate the underlying mechanism of roscovitine-induced inhibition of microglia activation and monocyte infiltration, we thus investigated the effect of roscovitine on NFκB-S276 phosphorylation. Following SE, NFκB-S276 phosphorylation was apparently increased in most of the activated IB4 microglia and CD68 cells, while it was rarely observed in control animals (Figure 4A,B). However, roscovitine did not affect p65 NFκB-S276 phosphorylation in activated IB4 microglia and CD68 cells (Figure 4A,C). These findings indicate that roscovitine may attenuate microglia activation and monocyte infiltration independent of NFκB-S276 phosphorylation.

### 3.5. The Effect of Roscovitine on p38 MAPK Activation in Activated Microglia and Monocytes

**Figure 5:**
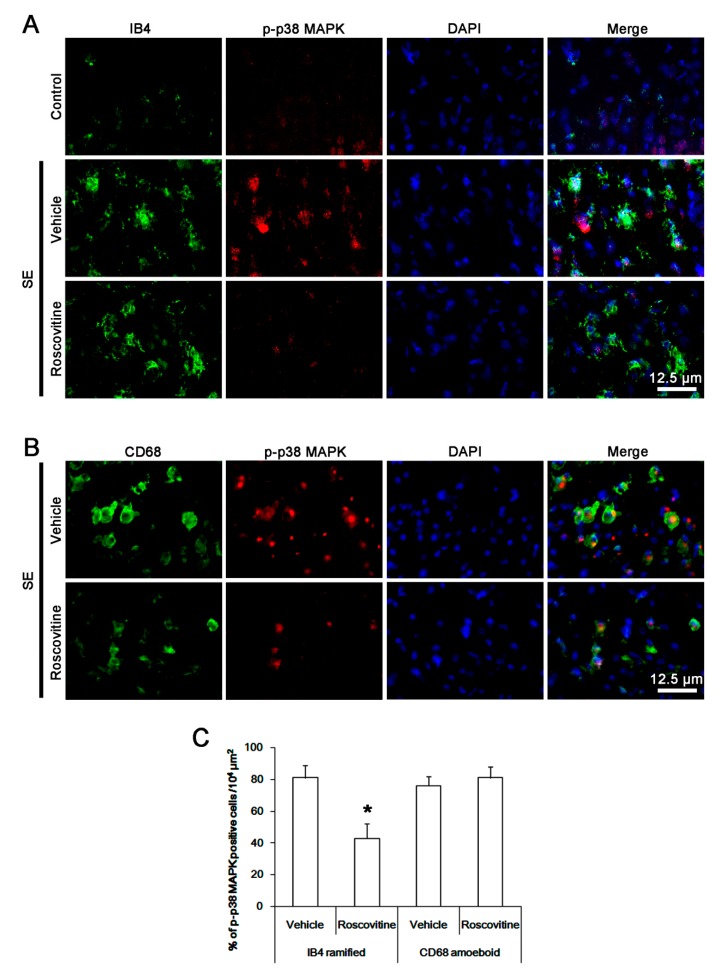
**The effect of roscovitine on p38 MAPK phosphorylation in microglia and monocyte following SE**. p38 MAPK phosphorylation is increased in resident IB4 microglia following SE, which is abrogated by roscovitine. CD68 amoeboid cells also show the up-regulated p38 MAPK phosphorylation, while roscovitine does not affect it. (A,B) Representative images for p38 MAPK phosphorylation in IB4 microglia and CD68 cells, respectively. (C) Quantification of the effect of roscovitine on p38 MAPK phosphorylation in IB4 microglia and CD68 cells following SE. Error bars indicate SEM (* p < 0.05 vs. vehicle-treated animals; *n* = 7, respectively).

p38 MAPK is also involved in pro-inflammatory cytokine/chemokine production and monocyte infiltration into the brain and the resident microglia activation [25]. Since roscovitine significantly inhibited p38 MAPK activity in microglia [26], it is likely that roscovitine may also ameliorate microglia activation and monocyte infiltration by inhibiting p38 MAPK signaling cascade. Following SE, p38 MAPK phosphorylation was increased in activated IB4 microglia, which was abrogated by roscovitine (*p* < 0.05 vs. vehicle, Student *t*-test, *n* = 7, respectively; Figure 5A,C). CD68 amoeboid cells also showed the phospho-p38 MAPK signals. However, roscovitine did not affect p38 MAPK phosphorylation in these cells (Figure 5B,C). These findings indicate that roscovitine may abolish p38 MAPK phosphorylation in activated IB4 microglia, but not in infiltrating monocytes, following SE. 

### 3.6. The Effect of SB202190 on Microglia Activation and Monocyte Infiltration Following SE

**Figure 6:**
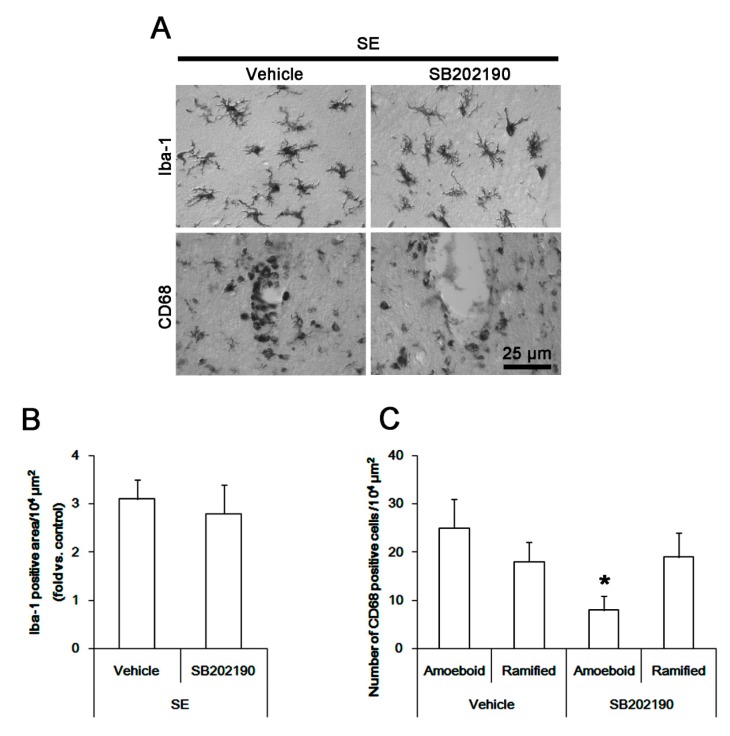
**The effect of SB202190 on microglia activation and monocyte infiltration in FPC following SE**. Although SB202190 does not influence Iba-1 microglia transformation, it reduces the number of CD68 amoeboid cells without altering that of CD68 hyper-ramified cells following SE. (A) Representative images for Iba-1 and CD68 positive cells. (B,C) Quantification of the effect of SB202190 on Iab-1 positive area (**B**) and the number of CD68 amoeboid and ramified cells (**C**) following SE. Error bars indicate SEM (* p < 0.05 vs. control- or vehicle-treated animals; *n* = 7, respectively).

**Figure 7:**
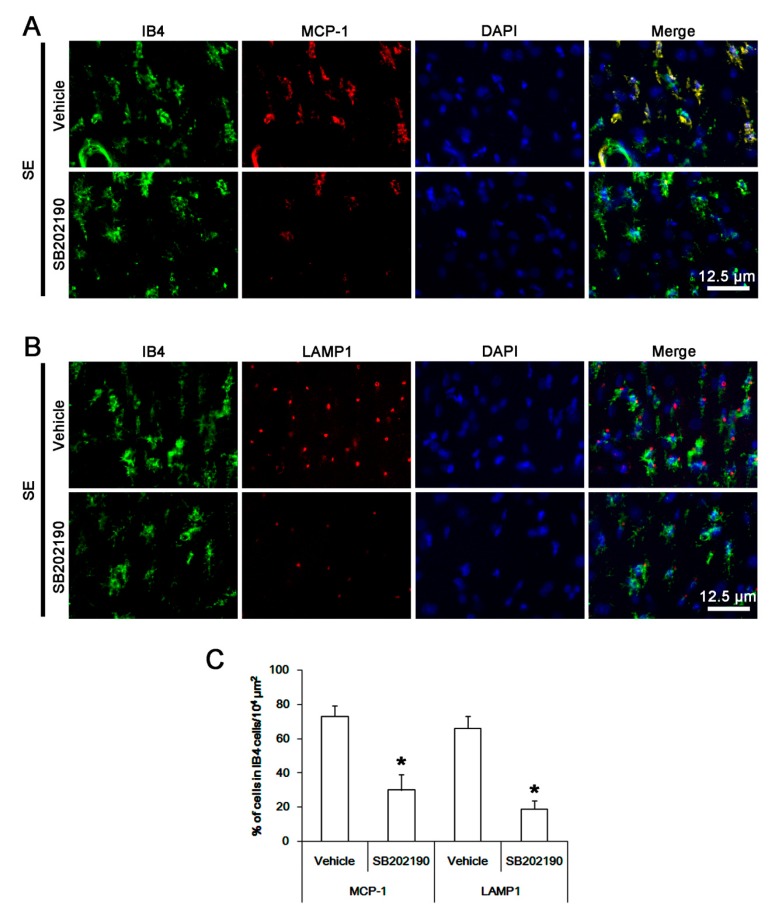
**The effects of SB202190 on MCP-1 induction and phagocytosis of resident microglia after SE**. As compared to vehicle, SB202190 diminishes MCP-1 and LAMP1 expressions in IB4 microglia following SE. (**A**,**B**) Representative images for MCP-1 and LAMP1 expressions in IB4 microglia. (**C**) Quantification of the effects of SB202190 on MCP-1 and LAMP1 expressions in IB4 microglia following SE. Error bars indicate SEM (* *p* < 0.05 vs. vehicle-treated animals; *n* = 7, respectively).

To confirm the role of p38 MAPK in microglia activation and monocyte infiltration, we applied SB202190 prior to SE induction. As compared to the vehicle, SB202190 did not affect the Iba-1 microglia transformation (Figure 6B). However, it led to ~ 32% reductions in the number of CD68 amoeboid cells without altering that of CD68 hyper-ramified cells in the FPC (*p* < 0.05 vs. vehicle, Student *t*-test, *n* = 7, respectively; Figure 6C). Furthermore, SB202190 attenuated expressions of MCP-1 and LAMP1 in activated microglia in the FPC following SE (*p* < 0.05 vs. vehicle, Student *t*-test, *n* = 7, respectively; Figure 7A,C). Therefore, our findings suggest that p38 MAPK in activated IB4 microglia, but not in infiltrating monocytes, may be one of the down-stream molecules for anti-neuroinflammatory activity of roscovitine, which would suppress phagocytosis and MCP-1 synthesis, regardless of the morphogenesis of activated microglia.

## 4. Discussion

The major findings in the present study are that roscovitine attenuated SE-induced microglia activation and monocyte infiltration in the FPC, accompanied by reducing p38 MAPK phosphorylation. Furthermore, SB202190, a p38 MAPK inhibitor, diminished MCP-1 and LMAP1 expressions in activated microglia and monocyte infiltration in this region following SE. Thus, our findings indicate that roscovitine may abolish SE-induced neuroinflammation via inhibition of p38 MAPK-mediated signaling pathway.

Infiltrating leukocytes are involved in cell injury by generation of reactive oxygen species, proteolytic enzymes and proinflammatory cytokines/chemokines [27,28]. Therefore, inhibition of leukocyte infiltration may be one of the therapeutic strategies for preventing secondary complication in various neurological diseases. CD68 is a marker of blood-derived monocytes as well as fully activated microglia, which have the phagocytic ability [29]. Furthermore, IB4 and Iba-1 are also markers for resident quiescent, activated microglia and infiltrating monocytes [30,31,32]. Therefore, it is difficult to distinguish between activated microglia and infiltrating monocytes using traditional microglia/macrophage markers. However, it has been widely considered that CD68 infiltrating monocyte shows a large, round morphology with a smooth cell surface. In contrast, activated resident microglia have the irregularly shaped soma and blunted processes, since resident quiescent microglia become activated quickly accompanying retraction of ramified thin, long processes and transformation into amoeboid cells [33]. In the present study, Iba-1 or IB4 microglia showed hypertrophic morphologies with irregularly shaped soma and blunted processes that were covered by a lot of thorny spine following SE. In contrast, CD68 cells showing amoeboid or round shapes with smooth cell surface appeared in perivascular brain parenchyma in post-SE animals. As described above, it is likely that CD68 amoeboid/round cells may be blood-derived monocytes, while Iba-1 or IB4 hypertrophic cells with blunted processes and CD68 hyper-ramified cells may be activated resident microglia. Interestingly, the present data demonstrate that roscovitine abrogated MCP-1 induction in activated IB4 microglia without affecting microglia transformation following SE. These findings indicate that roscovitine may inhibit MCP-1 synthesis in activated microglia, while it did not affect morphogenesis of activated microglia. Furthermore, roscovitine decreased the number of CD68 infiltrating monocytes in the brain parenchyma without changing CCR2 expression in CD68 cells following SE. Therefore, our findings suggest that roscovitine may abrogate SE-induced monocyte infiltration mediated by MCP-1 released from activated microglia.

In the present study, roscovitine also ameliorated LAMP1 expression in activated IB4 microglia without affecting microglia transformation following SE. Since phagocytic ability in microglia correlates with protein levels of LAMP1 [34,35], these findings indicate that roscovitine may inhibit phagocytosis in activated microglia, while it could not influence LAMP1 expressions in CD68 cells following SE. Thus, our findings suggest that roscovitine may inhibit phagocytic ability of activated microglia, but not that of monocytes, and that roscovitine may influence the activity of resident microglia rather than blood-derived monocytes. 

p65-Ser276 NFκB phosphorylation enhances its transactivation potential and interaction with cAMP response element-binding (CREB)-binding protein, which is important for the microglia activation [36,37]. In addition, p65-Ser276 NFκB phosphorylation modulates TNF-α expression and MCP-1 in macrophages [23]. In the present study, SE led to the increased NFκB-S276 phosphorylation in most of the activated IB4 microglia and CD68 cells. However, roscovitine did not affect p65 NFκB-S276 phosphorylation in these cells. These findings are consistent with a previous study demonstrating that roscovitine does not affect the NF-κB activation cascade such as an inhibitor of κB (IκB) kinase activity, IκB-α degradation and p65 translocation [38]. Since inhibition of p65-Ser276 NFκB phosphorylation effectively attenuates microglia transformation to amoeboid shape induced by SE [24], our findings indicate that the NFκB-mediated signaling pathway may regulate the morphogenesis of activated microglia rather than MCP-1 synthesis or phagocytosis of microglia. 

Since CDK5 is one of the serine/threonine cyclin-dependent CDKs and roscovitine is a potential CDK5 inhibitor, the anti-inflammatory properties of roscovitine is relevant to the inhibition of microglia proliferation [14]. However, roscovitine also affects the activities of Cdc2, CDK2, CDK9 and protein kinase A [17,38,39]. Furthermore, CDK5 is the upstream regulator of phosphorylation of p38 MAPK in microglia [26]. In the present study, roscovitine decreased up-regulation of p38 MAPK phosphorylation in activated IB4 microglia without affecting microglia transformation following SE. Similar to roscovitine, SB201290 attenuated CD68 monocyte infiltration and the increases in MCP-1 and LAMP1 expressions in activated microglia without altering the morphogenesis of activated microglia. Since p38 MAPK inhibitor suppresses phagocytosis and MCP-1 synthesis in microglia [40,41], our findings indicate that roscovitine-induced inhibition of p38 MAPK activity may ameliorate MCP-1 synthesis and phagocytosis in activated microglia following SE. With respect to previous reports demonstrating anti-microglia-related neuroinflammatory effects of roscovitine in various harmful conditions, such as brain trauma, tissue graft and ischemia [13,14,42], our findings propose that inhibition of the association between CDK5 and the p38 MAPK signaling pathway may present a potential therapeutic target for diminishing neuroinflammatory responses.

## 5. Conclusions

To the best of our knowledge, the present data demonstrate for the first time the effects of roscovitine on microglia activation and monocyte infiltration induced by SE. Briefly, roscovitine attenuated SE-induces monocyte infiltration without vasogenic edema formation in the FPC, accompanied by reducing expressions of MCP-1 and LAMP1, and p38 MAPK phosphorylation, but not nuclear factor-κB-S276 phosphorylation. SB202190 also ameliorated monocyte infiltration and microglial expressions of MCP-1 and LAMP1 in the FPC following SE. Therefore, we propose the availability of roscovitine for anti-neuroinflammatory substance to inhibit microglial responses and monocyte infiltration in various neurological diseases including seizures. 

## Figures and Tables

**Table 1:** Primary antibodies and lectin used in the present study.

Antigen	Host	Manufacturer (Catalog Number)	Dilution Used
Iba-1	Rabbit	Biocare Medical (CP 290)	1:500
IB4		Vector (B-1205)	1:200
CD68	Mouse	Abcam (ab31630)	1:100
p-p38 MAPK	Rabbit	Abbiotec (# 251256)	1:200
NFκB-S276	Rabbit	Abcam (ab106129)	1:100
LAMP1	Rabbit	Abcam (ab24170)	1:100
MCP-1	Mouse	Abcam (ab25124)	1:100
CCR2	Rabbit	Abcam (ab227015)	1:100

## References

[1] De Simoni M.G., Perego C., Ravizza T., Moneta D., Conti M., Marchesi F., De Luigi A., Garattini S., Vezzani A. (2000). Inflammatory cytokines and related genes are induced in the rat hippocampus by limbic status epilepticus. Eur. J. Neurosci..

[2] Plata-Salamán C.R., Ilyin S.E., Turrin N.P., Gayle D., Flynn M.C., Romanovitch A.E., Kelly M.E., Bureau Y., Anisman H., McIntyre D.C. (2000). Kindling modulates the IL-1β system, TNF-alpha, TGF-β1, and neuropeptide mRNAs in specific brain regions. Brain Res. Mol. Brain Res..

[3] Rizzi M., Perego C., Aliprandi M., Richichi C., Ravizza T., Colella D., Velískŏvá J., Moshé S.L., De Simoni M.G., Vezzani A. (2003). Glia activation and cytokine increase in rat hippocampus by kainic acid-induced status epilepticus during postnatal development. Neurobiol. Dis..

[4] Vezzani A., Conti M., De Luigi A., Ravizza T., Moneta D., Marchesi F., De Simoni M.G. (1999). Interleukin-1beta immunoreactivity and microglia are enhanced in the rat hippocampus by focal kainate application: Functional evidence for enhancement of electrographic seizures. J. Neurosci..

[5] Vezzani A., Moneta D., Conti M., Richichi C., Ravizza T., De Luigi A., De Simoni M.G., Sperk G., Andell-Jonsson S., Lundkvist J. (2000). Powerful anticonvulsant action of IL-1 receptor antagonist on intracerebral injection and astrocytic overexpression in mice. Proc. Natl. Acad. Sci. USA.

[6] DeLorenzo R.J., Pellock J.M., Towne A.R., Boggs J.G. (1995). Epidemiology of status epilepticus. J. Clin. Neurophysiol..

[7] Kim J.E., Ryu H.J., Yeo S.I., Kang T.C. (2010). P2X7 receptor regulates leukocyte infiltrations in rat frontoparietal cortex following status epilepticus. J. Neuroinflamm..

[8] Jo S.M., Ryu H.J., Kim J.E., Yeo S.I., Kim M.J., Choi H.C., Song H.K., Kang T.C. (2011). Up-regulation of endothelial endothelin-1 expression prior to vasogenic edema formation in the rat piriform cortex following status epilepticus. Neurosci. Lett..

[9] Ravizza T., Gagliardi B., Noé F., Boer K., Aronica E., Vezzani A. (2008). Innate and adaptive immunity during epileptogenesis and spontaneous seizures: Evidence from experimental models and human temporal lobe epilepsy. Neurobiol. Dis..

[10] Carr M.W., Roth S.J., Luther E., Rose S.S., Springer T.A. (1994). Monocyte chemoattractant protein 1 acts as a T-lymphocyte chemoattractant. Proc. Natl. Acad. Sci. USA.

[11] Fuentes M.E., Durham S.K., Swerdel M.R., Lewin A.C., Barton D.S., Megill J.R., Bravo R., Lira S.A. (1995). Controlled recruitment of monocytes and macrophages to specific organs through transgenic expression of monocyte chemoattractant protein-1. J. Immunol..

[12] Leitch A.E., Riley N.A., Sheldrake T.A., Festa M., Fox S., Duffin R., Haslett C., Rossi A.G. (2010). The cyclin-dependent kinase inhibitor R-roscovitine down-regulates Mcl-1 to override pro-inflammatory signalling and drive neutrophil apoptosis. Eur. J. Immunol..

[13] Hilton G.D., Stoica B.A., Byrnes K.R., Faden A.I. (2008). Roscovitine reduces neuronal loss, glial activation, and neurologic deficits after brain trauma. J. Cereb. Blood Flow Metab..

[14] Tomov N., Surchev L., Wiedenmann C., Döbrössy M., Nikkhah G. (2019). Roscovitine, an experimental CDK5 inhibitor, causes delayed suppression of microglial, but not astroglial recruitment around intracerebral dopaminergic grafts. Exp. Neurol..

[15] Vita M., Abdel-Rehim M., Olofsson S., Hassan Z., Meurling L., Sidén A., Sidén M., Pettersson T., Hassan M. (2005). Tissue distribution, pharmacokinetics and identification of roscovitine metabolites in rat. Eur. J. Pharm. Sci..

[16] Sallam H., Jimenez P., Song H., Vita M., Cedazo-Minguez A., Hassan M. (2008). Age-dependent pharmacokinetics and effect of roscovitine on Cdk5 and Erk1/2 in the rat brain. Pharmacol. Res..

[17] Hyun H.W., Min S.J., Kim J.E. (2017). CDK5 inhibitors prevent astroglial apoptosis and reactive astrogliosis by regulating PKA and DRP1 phosphorylations in the rat hippocampus. Neurosci. Res..

[18] Kim J.E., Kang T.C. (2018). Nucleocytoplasmic p27(Kip1) export is required for ERK1/2-mediated reactive astroglial proliferation following status epilepticus. Front. Cell. Neurosci..

[19] Kim D.S., Min S.J., Kim M.J., Kim J.E., Kang T.C. (2016). Leptomycin B ameliorates vasogenic edema formation induced by status epilepticus via inhibiting p38 MAPK/VEGF pathway. Brain Res..

[20] Streit W.J., Walter S.A., Pennell N.A. (1999). Reactive microgliosis. Prog. Neurobiol..

[21] Ramprasad M.P., Terpstra V., Kondratenko N., Quehenberger O., Steinberg D. (1996). Cell surface expression of mouse macrosialin and human CD68 and their role as macrophage receptors for oxidized low density lipoprotein. Proc. Natl. Acad. Sci. USA.

[22] Fu H., Liu B., Frost J.L., Hong S., Jin M., Ostaszewski B., Shankar G.M., Costantino I.M., Carroll M.C., Mayadas T.N. (2012). Complement component C3 and complement receptor type 3 contribute to the phagocytosis and clearance of fibrillar Aβ by microglia. Glia.

[23] Furusawa J., Funakoshi-Tago M., Tago K., Mashino T., Inoue H., Sonoda Y., Kasahara T. (2009). Licochalcone A significantly suppresses LPS signaling pathway through the inhibition of NF-κB p65 phosphorylation at serine 276. Cell Signal..

[24] Lee S.K., Kim J.E., Kim Y.J., Kim M.J., Kang T.C. (2014). Hyperforin attenuates microglia activation and inhibits p65-Ser276 NFκB phosphorylation in the rat piriform cortex following status epilepticus. Neurosci. Res..

[25] Morganti J.M., Goulding D.S., Van Eldik L.J. (2019). Deletion of p38α MAPK in microglia blunts trauma-induced inflammatory responses in mice. J. Neuroinflamm..

[26] Fang-Hu, Zhang H.H., Yang B.X., Huang J.L., Shun J.L., Kong F.J., Peng-Xu, Chen Z.G., Lu J.M. (2015). Cdk5 contributes to inflammation-induced thermal hyperalgesia mediated by the p38 MAPK pathway in microglia. Brain Res..

[27] Dinkel K., Dhabhar F.S., Sapolsky R.M. (2004). Neurotoxic effects of polymorphonuclear granulocytes on hippocampal primary cultures. Proc. Natl. Acad. Sci. USA.

[28] Kielian T., Barry B., Hickey W.F. (2001). CXC chemokine receptor-2 ligands are required for neutrophil-mediated host defense in experimental brain abscesses. J. Immunol..

[29] Streit WJ., Kettenmann H., Ransom B.R. (2005). Microglial cells. Neuroglia.

[30] Morioka T., Kalehua A.N., Streit W.J. (2005). The microglial reaction in the rat dorsal hippocampus following transient forebrain ischemia. J. Cereb. Blood Flow Metab..

[31] Imai Y., Ibata I., Ito D., Ohsawa K., Kohsaka S. (1996). A novel gene iba1 in the major histocompatibility complex class III region encoding an EF hand protein expressed in a monocytic lineage. Biochem. Biophys. Res. Commun..

[32] Ito D., Imai Y., Ohsawa K., Nakajima K., Fukuuchi Y., Kohsaka S. (1998). Microglia-specific localisation of a novel calcium binding protein, Iba1. Brain Res. Mol. Brain Res..

[33] Matsumoto H., Kumon Y., Watanabe H., Ohnishi T., Shudou M., Ii C., Takahashi H., Imai Y., Tanaka J. (2007). Antibodies to CD11b, CD68, and lectin label neutrophils rather than microglia in traumatic and ischemic brain lesions. J. Neurosci. Res..

[34] Rojanathammanee L., Murphy E.J., Combs C.K. (2011). Expression of mutant alpha-synuclein modulates microglial phenotype In Vitro. J. Neuroinflamm..

[35] Tanaka Y., Matsuwaki T., Yamanouchi K., Nishihara M. (2013). Increased lysosomal biogenesis in activated microglia and exacerbated neuronal damage after traumatic brain injury in progranulin-deficient mice. Neuroscience.

[36] Roy A., Fung Y.K., Liu X., Pahan K. (2006). Up-regulation of microglial CD11b expression by nitric oxide. J. Biol. Chem..

[37] Zhong L.M., Zong Y., Sun L., Guo J.Z., Zhang W., He Y., Song R., Wang W.M., Xiao C.J., Lu D. (2012). Resveratrol inhibits inflammatory responses via the mammalian target of rapamycin signaling pathway in cultured LPS-stimulated microglial cells. PLoS ONE.

[38] Berberich N., Uhl B., Joore J., Schmerwitz U.K., Mayer B.A., Reichel C.A., Krombach F., Zahler S., Vollmar A.M., Fürst R. (2011). Roscovitine blocks leukocyte extravasation by inhibition of cyclin-dependent kinases 5 and 9. Br. J. Pharmacol..

[39] Meijer L., Borgne A., Mulner O., Chong J.P., Blow J.J., Inagaki N., Inagaki M., Delcros J.G., Moulinoux J.P. (1997). Biochemical and cellular effects of roscovitine, a potent and selective inhibitor of the cyclin-dependent kinases cdc2, cdk2 and cdk5. Eur. J. Biochem..

[40] Katayama T., Kobayashi H., Okamura T., Yamasaki-Katayama Y., Kibayashi T., Kimura H., Ohsawa K., Kohsaka S., Minami M. (2012). Accumulating microglia phagocytose injured neurons in hippocampal slice cultures: Involvement of p38 MAP kinase. PLoS ONE.

[41] Zhou Y., Ling E.A., Dheen S.T. (2007). Dexamethasone suppresses monocyte chemoattractant protein-1 production via mitogen activated protein kinase phosphatase-1 dependent inhibition of Jun N-terminal kinase and p38 mitogen-activated protein kinase in activated rat microglia. J. Neurochem..

[42] Zhang Q., Chen C., Lü J., Xie M., Pan D., Luo X., Yu Z., Dong Q., Wang W. (2009). Cell cycle inhibition attenuates microglial proliferation and production of IL-1beta, MIP-1alpha, and NO after focal cerebral ischemia in the rat. Glia.

